# Editorial: Community series in reducing adverse effects of cancer immunotherapy, volume III

**DOI:** 10.3389/fimmu.2026.1980572

**Published:** 2026-09-09

**Authors:** Leonardo Vinícius Barbosa, Lara Luisa Valerio de Mello Braga, Lucas Antonio Duarte Nicolau, Claire J. Han, Daniele Maria-Ferreira, Cleber Machado de Souza

**Affiliations:** 1 Faculdades Pequeno Príncipe, Curitiba, Brazil; 2 Instituto de Pesquisa Pelé Pequeno Príncipe, Curitiba, Brazil; 3 Laboratory of Inflammation and Translational Gastroenterology (LIGAT), Post-graduation Program in Biotechnology (PPGBIOTEC), Parnaíba Delta Federal University, Parnaíba, Brazil; 4 Center for Healthy Aging, Self-Management and Complex Care, College of Nursing, The Ohio State University, Columbus, OH, United States; 5 The James: Comprehensive Cancer Treatment and Research Center, The Ohio State University, Columbus, OH, United States

**Keywords:** adverse effects, cancer immunotherapy, cancer treatment, immune-related adverse events, toxicity management, treatment related toxicity

Chemotherapy, radiotherapy, and immunotherapy have substantially improved cancer treatment outcomes and survival. At the same time, these therapies can cause a broad spectrum of adverse effects that may impair quality of life, disrupt treatment continuity, and, in some cases, limit the therapeutic options available to patients. In this context, understanding treatment-related toxicities has become increasingly important as cancer therapies continue to evolve.

As the third volume of the *“Reducing Adverse Effects of Cancer Immunotherapy”* Community Series, this Research Topic brings together studies addressing the diverse manifestations of treatment-related toxicity, with particular emphasis on immune-related adverse events (irAEs). The contributions range from individual case reports to systematic reviews, meta-analyses, retrospective and prospective studies, and real-world pharmacovigilance analyses. Collectively, they explore the clinical presentation, underlying mechanisms, risk factors, prediction, monitoring, and management of toxicities associated with contemporary cancer therapies.

## Clinical manifestations and management of treatment-related toxicities

Several contributions focused on uncommon but potentially severe toxicities associated with immune-based cancer therapies. In patients with urothelial bladder cancer and chronic kidney disease, distinguishing immune-related adverse events from complications of pre-existing disease can be particularly challenging. A case of nivolumab-associated muscular and endocrine toxicity illustrates the importance of close multidisciplinary monitoring, prompt recognition of immune-mediated complications, and careful initiation and tapering of corticosteroids (Najmrocka et al.).

Hematologic complications were also discussed. A case of pembrolizumab-induced aplastic anemia in metastatic cervical cancer was associated with an HLA-A*0201 haplotype and a newly identified DNMT3A mutation related to clonal hematopoiesis. These findings provide potential clues to the genetic background underlying immune-related aplastic anemia and highlight the complexity of predicting rare hematologic toxicities (Takahashi et al.).

Other studies examined immune-mediated dermatologic and mucosal complications. A systematic review of 116 cases of immune checkpoint inhibitor-induced bullous pemphigoid found that severity-based treatment was generally effective and that immune checkpoint inhibitor rechallenge could be considered in selected patients. Recurrence occurred in 22.2% of rechallenged patients, while severe disease, mucosal involvement, and advanced age were associated with greater clinical risk (Chang et al.). Similarly, a review of 18 cases of pembrolizumab-associated immune-related oral mucositis found that symptoms typically developed after prolonged treatment and most presented as painful ulcers or erosions. Corticosteroid therapy was effective in most cases, with favorable recovery and successful rechallenge possible in selected patients (Yu et al.).

The severity of immune-mediated skin toxicity was further illustrated by a case of severe toxic epidermal necrolysis following the first infusion of sintilimab in a patient with metastatic gastric cancer. Recovery was achieved through a multidisciplinary approach involving immune checkpoint inhibitor withdrawal, immunosuppression, wound care, infection control, and nutritional support, underscoring the importance of early recognition and coordinated management of life-threatening toxicities (Zhao et al.). A retrospective analysis of 31 reported cases of nivolumab-associated Stevens-Johnson syndrome/toxic epidermal necrolysis similarly demonstrated the severity of these rare events. The median onset was 5.5 weeks, and although most patients improved with treatment, mortality reached 22.6% (Li et al.).

Drug-related toxicity may also result from interactions between anticancer agents and concomitant medications. A case report described grade IV thrombocytopenia associated with the concomitant use of selpercatinib and voriconazole in a patient with advanced lung cancer. Platelet counts recovered after treatment discontinuation, allowing selpercatinib to be successfully reintroduced at a reduced dose. The report highlights a potential CYP3A4-mediated interaction and the need for careful monitoring and dose adjustment (Tang et al.).

A review of 39 reported cases of rituximab-induced serum sickness provided another example of the heterogeneous presentation of treatment-related toxicity. Fever, rash, and arthralgia or arthritis were the predominant manifestations and were frequently accompanied by elevated inflammatory markers and hypocomplementemia. Most patients recovered following drug withdrawal and corticosteroid therapy, whereas rechallenge was frequently associated with recurrence (Huang et al.).

## Organ-specific toxicity and potential mechanisms

Beyond individual clinical manifestations, several contributions examined the mechanisms underlying immune-related toxicities and potential biomarkers for their early recognition.

Immune checkpoint inhibitor-associated myocarditis remains a rare but potentially severe complication. A review by Huang et al. examined its proposed mechanisms and emerging biomarkers, highlighting CD8+ T-cell expansion, loss of regulatory immune cells, macrophage activation, and interactions among multiple immune cell populations as possible contributors to myocardial injury. Candidate biomarkers and therapeutic targets may help improve early diagnosis and cardioprotection.

Pulmonary and renal toxicities were addressed from a complementary perspective. A review of bronchoalveolar lavage fluid in immune checkpoint inhibitor-associated pneumonitis discussed its potential value for cytologic, molecular, and multi-omics analyses. BALF lymphocytosis and biomarkers such as CCL18, IL-6, and IP-10 may contribute to diagnosis and disease monitoring, although standardized sampling and interpretation remain necessary (Linpeng et al.).

Renal immune-related adverse events were examined in the context of immune checkpoint inhibitor therapy, with particular attention to immunologic mechanisms, susceptibility genes, biomarkers, and management strategies. The review also considered patients with chronic kidney disease, renal transplantation, and end-stage renal disease, for whom individualized surveillance may be especially important (Lv et al.).

Other studies extended the discussion of organ-specific toxicity to less frequently recognized complications. In a retrospective analysis of 982 patients receiving immune checkpoint inhibitors, elevated pretreatment serum amylase and immune-related adverse events affecting other organs were identified as independent risk factors for immune-related pancreatic injury. Interestingly, patients who developed pancreatic injury had better overall survival, suggesting that systematic monitoring may facilitate earlier identification of this complication (Akazawa et al.).

The effects of immune checkpoint blockade on bone health were also reviewed. Although PD-1/PD-L1 inhibitors may have bone-protective effects under some circumstances, their use has also been associated with osteoporosis and fragility fractures. The authors propose considering these events within the broader spectrum of potential irAEs and recommend risk-stratified surveillance of bone health (Wang et al.).

## Predicting toxicity and identifying patients at risk

Early identification of patients at increased risk of treatment-related toxicity is an important step toward more individualized cancer care. Several contributions explored clinical, laboratory, microbiological, and computational approaches for this purpose.

A study involving 32 patients with lung cancer receiving immune checkpoint inhibitors investigated longitudinal changes in gut microbiota and metabolites in relation to immune-related adverse events. Distinct microbial and metabolic profiles were observed at baseline and around the onset of irAEs. A machine learning model showed promising predictive performance, suggesting that integrating microbiome and metabolomic information may contribute to earlier identification of patients at risk (Han et al.).

Potential predictors were also explored at the hematologic level. A systematic review and meta-analysis including 60 studies and 16,736 patients evaluated peripheral blood cell counts as predictors of irAEs. Higher baseline lymphocyte and eosinophil counts, together with lower neutrophil counts, neutrophil-to-lymphocyte ratios, and platelet-to-lymphocyte ratios, were associated with an increased risk of irAEs. These readily available parameters may therefore have value as accessible biomarkers for risk stratification (Zhang et al.).

Risk factors for cutaneous irAEs were examined in a scoping review of 50 studies involving 198,514 patients. Sixty-eight potential risk factors were identified, including demographic characteristics, treatment regimen, tumor type, and inflammatory biomarkers. However, the authors noted substantial limitations in the quality and consistency of the available evidence, reinforcing the need for prospective validation of proposed predictors (Su et al.).

Machine learning was also applied to predicting cytokine release syndrome following CAR-T therapy. Among the evaluated approaches, XGBoost showed the best performance, with oxygen saturation, D-dimer, diastolic blood pressure, and INR emerging as key predictors. The model also demonstrated promising performance in an independent CAR-T cohort, supporting further evaluation of computational approaches for early risk assessment (Yu et al.).

Treatment timing may represent another modifiable determinant of toxicity. In a retrospective study of 93 pediatric patients with acute lymphoblastic leukemia, concurrent intrathecal chemotherapy during blinatumomab infusion was not associated with increased neurotoxicity overall. However, administration on day 1 was independently associated with a higher risk of neurotoxicity, suggesting that treatment scheduling may influence neurological safety (Liu et al.).

Cognitive effects of cancer treatment were investigated in a propensity score-matched prospective study comparing patients receiving chemotherapy with or without immune checkpoint inhibitors. Combined treatment was associated with greater cognitive impairment at one year. However, the use of screening tools and self-reported measures limits the strength of these conclusions and highlights the need for comprehensive neuropsychological assessment in future studies (Jian et al.).

## Real-world safety and pharmacovigilance

Real-world pharmacovigilance analyses provide an additional perspective on treatment-related toxicity by capturing adverse events across broader patient populations and routine clinical settings.

Li et al. analyzed real-world safety data from VigiBase and FAERS for first-line enfortumab vedotin plus pembrolizumab. Among 2,590 reports, they identified several previously unlisted adverse events, including dehydration, cholestasis, ileus, and tumor lysis syndrome. Differences in adverse-event profiles according to sex and time to onset further illustrate the value of pharmacovigilance data for characterizing safety signals beyond those detected in clinical trials.

A FAERS-based analysis of pembrolizumab and nivolumab in esophageal cancer identified endocrine, hepatic, renal, gastrointestinal, cardiovascular, and pulmonary toxicities. Earlier onset was observed in older and female patients, while greater cumulative adverse-event burden and earlier onset were associated with higher mortality, suggesting that both the type and timing of adverse events may have prognostic relevance (Chen et al.).

Treatment-specific safety profiles were further illustrated by a pharmacovigilance analysis comparing nivolumab and cetuximab in head and neck cancer. Nivolumab showed stronger associations with immune-mediated, hepatobiliary, and endocrine events, whereas cetuximab was more strongly associated with dermatologic and metabolic toxicities. These differences support the use of therapy-specific surveillance strategies (Tang et al.).

In hepatocellular carcinoma, real-world data on durvalumab plus tremelimumab showed strong signals for immune-mediated hepatic disorders, enterocolitis, and cytokine release syndrome. Most adverse events occurred within 30 days, and several immune-mediated toxicities were associated with drug-related mortality, reinforcing the importance of close monitoring during the early treatment period (Cheng et al.).

Cardiovascular safety was another recurring theme. A pharmacovigilance analysis of 4,797 reports involving sipuleucel-T identified cardiovascular adverse events in 15.5% of reports, with hypertension, thromboembolism, cardiac failure, arrhythmias, and myocardial infarction among the main signals. Most events occurred within the first month, supporting early cardiovascular monitoring (Liu et al.).

Similarly, a study using three international databases evaluated cardiac failure associated with novel antineoplastic agents in breast cancer. Trastuzumab showed consistently strong reporting signals, while HER2-targeted therapies generally showed higher signals than conventional chemotherapy. These findings emphasize the importance of individualized cardiovascular risk assessment when selecting and monitoring anticancer treatments (Li et al.).

A separate FAERS analysis characterized adverse events associated with pembrolizumab in cervical cancer. Most events were reported within 3–6 months of treatment, with hematologic, endocrine, dermatologic, neurologic, gastrointestinal, urinary, and reproductive toxicities among the main signals. The broad range of affected organ systems reinforces the need for systematic, organ-specific monitoring during treatment (Zhang et al.).

## Strategies to reduce treatment-related toxicity

Several contributions addressed an equally important question: how treatment strategies can be modified to reduce toxicity without compromising oncologic outcomes.

A systematic review and meta-analysis of 27 studies including 7,007 pediatric cancer patients examined concomitant medications associated with cisplatin-induced hearing loss. Vincristine, furosemide, aminoglycosides, and vancomycin were consistently associated with increased risk, emphasizing the importance of carefully evaluating concomitant medication use during cisplatin treatment (Jager et al.).

Treatment duration was another modifiable factor. A systematic review and meta-analysis involving 16,420 patients with stage II-III colorectal cancer compared three versus six months of oxaliplatin-based adjuvant chemotherapy. Three months of treatment were associated with less severe peripheral neuropathy and improved treatment completion, although six months remained preferable for patients with stage III disease receiving FOLFOX (Wu et al.).

Radiotherapy schedules were similarly evaluated in early-stage breast cancer. Across eight studies involving 5,495 patients, hypofractionated radiotherapy provided comparable disease-free survival and lymphedema rates to conventional fractionation, with a possible reduction in acute skin toxicity. However, substantial heterogeneity across studies warrants cautious interpretation of these findings (Talal et al.).

The potential for reducing unnecessary treatment exposure was also examined in early-stage cervical cancer. A systematic review and meta-analysis of eight studies involving 1,396 patients found no significant reduction in recurrence or mortality with postoperative adjuvant therapy following radical hysterectomy. These findings support a selective, risk-adapted approach to adjuvant treatment to avoid unnecessary toxicity in patients who may not derive sufficient benefit (Wang et al.).

## Treatment safety beyond toxicity

The impact of cancer therapy extends beyond conventional organ-specific adverse events. A cross-sectional study involving 232 patients with advanced head and neck cancer found lower levels of depression among those receiving chemoradiotherapy combined with immune checkpoint blockade compared with those receiving chemoradiotherapy alone. Although these findings do not establish causality, they highlight the importance of considering psychological outcomes alongside treatment safety and efficacy (Zhang et al.).

A case report of advanced thymic squamous cell carcinoma described a multimodal regimen combining tislelizumab, lenvatinib, polymeric micellar paclitaxel, and carboplatin. Sustained tumor reduction was accompanied by improvements in nutritional status and quality-of-life measures without significant immune-related adverse events, illustrating how individualized treatment strategies may balance disease control, tolerability, and patient-centered outcomes (Chen et al.).

Finally, a systematic review and meta-analysis of 11 randomized controlled trials involving 748 patients evaluated modified Xiaoyao-san for postoperative depression in breast cancer. The combination of modified Xiaoyao-san and antidepressants improved depressive symptoms compared with antidepressants alone, although additional high-quality trials are needed to confirm these findings and clarify their clinical relevance (Huang et al.).

## Conclusions

This Research Topic brings together a broad range of studies illustrating the increasingly diverse spectrum of adverse effects associated with cancer therapies. While immune checkpoint inhibitors and other immunotherapeutic approaches remain a major focus, the contributions also show that toxicity remains a significant challenge across chemotherapy, radiotherapy, targeted therapies, and combination regimens. Taken together, the studies highlight three complementary priorities for improving treatment safety: recognizing toxicities as early as possible, identifying patients who may be particularly vulnerable, and developing individualized strategies to manage or prevent adverse events without compromising anticancer efficacy. The growing use of real-world pharmacovigilance, circulating biomarkers, microbiome-based approaches, and machine learning models may further strengthen this effort. At the same time, the clinical reports presented in this Research Topic emphasize that effective management often depends on timely recognition, individualized decision-making, and multidisciplinary care. Ultimately, reducing treatment-related toxicity is not simply a matter of managing adverse events after they occur. It requires integrating safety assessment throughout the entire treatment pathway, from risk prediction and therapeutic selection to continuous monitoring and individualized management. Such an approach may help preserve both the effectiveness of modern cancer therapies and the quality of life of the patients who receive them.

We sincerely thank all authors, reviewers, and editors for their valuable contributions to this Research Topic and for advancing research aimed at making cancer treatment safer while preserving its therapeutic benefits.

